# Sequencing and Analysis of Globally Obtained Human Respiratory Syncytial Virus A and B Genomes

**DOI:** 10.1371/journal.pone.0120098

**Published:** 2015-03-20

**Authors:** Michael E. Bose, Jie He, Susmita Shrivastava, Martha I. Nelson, Jayati Bera, Rebecca A. Halpin, Christopher D. Town, Hernan A. Lorenzi, Daniel E. Noyola, Valeria Falcone, Giuseppe Gerna, Hans De Beenhouwer, Cristina Videla, Tuckweng Kok, Marietjie Venter, John V. Williams, Kelly J. Henrickson

**Affiliations:** 1 Midwest Respiratory Virus Program, Medical College of Wisconsin, Milwaukee, WI, USA; 2 J. Craig Venter Institute, Rockville, MD, United States of America; 3 Fogarty International Center, National Institutes of Health, Bethesda, MD, United States of America; 4 Departamento de Microbiología, Facultad de Medicina, Universidad Autónoma de San Luis Potosí, San Luis Potosí, Mexico; 5 Department of Virology, Institute for Medical Microbiology and Hygiene, University Medical Center Freiburg, Freiburg, Germany; 6 Laboratori Sperimentali di Ricerca, Fondazione IRCCS Policlinico San Matteo, Pavia, Italy; 7 Department of Microbiology, OLV Hospital Aalst, Aalst, Belgium; 8 Clinical Virology Laboratory, Centro de Educación Médica e Investigaciones Clínicas University Hospital, Buenos Aires, Argentina; 9 School of Molecular and Biomedical Science, University of Adelaide, Adelaide, Australia; 10 Zoonoses Research Unit, Department Medical Virology, University of Pretoria, Pretoria, South Africa; 11 Departments of Pediatrics and Pathology, Microbiology, and Immunology, Vanderbilt University School of Medicine, Nashville, TN, United States of America; University of Iowa, UNITED STATES

## Abstract

**Background:**

Human respiratory syncytial virus (RSV) is the leading cause of respiratory tract infections in children globally, with nearly all children experiencing at least one infection by the age of two. Partial sequencing of the attachment glycoprotein gene is conducted routinely for genotyping, but relatively few whole genome sequences are available for RSV. The goal of our study was to sequence the genomes of RSV strains collected from multiple countries to further understand the global diversity of RSV at a whole-genome level.

**Methods:**

We collected RSV samples and isolates from Mexico, Argentina, Belgium, Italy, Germany, Australia, South Africa, and the USA from the years 1998-2010. Both Sanger and next-generation sequencing with the Illumina and 454 platforms were used to sequence the whole genomes of RSV A and B. Phylogenetic analyses were performed using the Bayesian and maximum likelihood methods of phylogenetic inference.

**Results:**

We sequenced the genomes of 34 RSVA and 23 RSVB viruses. Phylogenetic analysis showed that the RSVA genome evolves at an estimated rate of 6.72 × 10^-4^ substitutions/site/year (95% HPD 5.61 × 10^-4^ to 7.6 × 10^-4^) and for RSVB the evolutionary rate was 7.69 × 10^-4^ substitutions/site/year (95% HPD 6.81 × 10^-4^ to 8.62 × 10^-4^). We found multiple clades co-circulating globally for both RSV A and B. The predominant clades were GA2 and GA5 for RSVA and BA for RSVB.

**Conclusions:**

Our analyses showed that RSV circulates on a global scale with the same predominant clades of viruses being found in countries around the world. However, the distribution of clades can change rapidly as new strains emerge. We did not observe a strong spatial structure in our trees, with the same three main clades of RSV co-circulating globally, suggesting that the evolution of RSV is not strongly regionalized.

## Introduction

Human respiratory syncytial virus (RSV) is the leading cause of respiratory tract illness in children globally, with nearly all children experiencing at least one infection by the age of two [1]. RSV can also regularly infect adults and causes severe illness in the elderly [2,3]. As a member of the *Paramyxoviridae* family, RSV is a non-segmented negative strand RNA virus whose genome contains ten genes that encode 11 proteins. RSV is divided into two distinct subtypes (A and B) and each have been divided into multiple genotypes that have been found to co-circulate [4,5]. Most genetic studies of RSV are focused on the attachment glycoprotein (G) gene which is the most variable and has been commonly used for genotyping [6–8]. The same genotypes have generally been shown to circulate globally [9–12].

When we started this study in 2009, there were relatively few sequences for RSV in GenBank. The majority were partial coding sequences, and the limited number of whole genome sequences were mainly from viruses collected more than 20 years prior or experimentally derived mutants. The lack of whole-genome data made it particularly difficult to design diagnostic tests for RSV capable of detecting the broad diversity of modern strains. To fill this void, we collected RSV viruses from Mexico, Argentina, Belgium, Italy, Germany, Australia, South Africa, and the USA from 1998–2010. From these isolates we sequenced the complete genomes of 57 RSV viruses (34 RSVA and 23 RSVB viruses). Since we started this study there have been parallel efforts to sequence RSV genomes, so we have included these additional data in our analyses. A phylogenetic analysis based on the whole genome, full G gene coding sequence (CDS), and the second variable region of the G gene identified multiple clades circulating globally.

## Materials and Methods

### Ethics Statement

Samples from the Virology Laboratory of CEMIC, University of Adelaide, University Medical Center Freiburg, and Onze Lieve Vrouw Ziekenhuis were collected for routine viral diagnostic testing and de-identified prior to shipment to the Medical College of Wisconsin. Samples from the Autonomous University of San Luis Potosí were collected with written informed consent as approved by the Research and Ethics Committee. Samples from the Fondazione IRCCS Policlinico San Matteo were collected with written informed consent as approved by the Bioethics Committee of the Fondazione IRCCS Policlinico San Matteo. Samples from the University of Pretoria were collected with written informed consent as approved by the Health Sciences Research Ethics Committee. Samples from the Vanderbilt Vaccine Clinic were collected with written informed consent as approved by the Committee for the Protection of Human Subjects of the Vanderbilt University Medical Center. At Children’s Hospital of Wisconsin and Froedtert Hospital samples were collected with written informed consent as approved by the Children’s Hospital of Wisconsin Human Research Review Board. For samples collected from minors written informed consent was obtained from their parent or guardian on their behalf. Some samples had been de-identified and where tested under a protocol approved by the Children’s Hospital of Wisconsin Human Research Review Board.

### Sample Collection and RSV Identification from Various Locations

#### The Autonomous University of San Luis Potosí (San Luis Potosí, Mexico)

Respiratory samples were collected as part of research projects carried out to analyze the epidemiology of viral respiratory infections and as part of a hospital-based infection control program; the research projects were approved by the corresponding Research and Ethics Committees. Samples were obtained by nasal wash or pharyngeal swab. Viral testing was carried out directly on respiratory samples. RSV was identified with a direct immunofluorescence assay.

#### Virology Laboratory of CEMIC (Buenos Aires, Argentina)

Nasopharyngeal aspirates or swabs were submitted for routine viral diagnostics in a transport media (Hanks plus 2% FCS, penicillin, streptomycin, and amphotericin B). Samples were processed for antigen detection on pelleted cells by indirect immunofluorescence with monoclonal antibodies against RSV, adenovirus, influenza A and B and parainfluenza (EMD Millipore, Billerica, MA, USA). A fluorescein labeled anti-mouse IgG was used (Sigma-Aldrich Corp., St. Louis, MO, USA). Readings were performed with a C. Zeiss microscope provided with epifluorescent equipment and a mercury lamp.

#### University of Adelaide, IMVS-SA Pathology (Adelaide, Australia)

Respiratory secretion specimens were submitted for routine viral diagnostics for seven viruses (adenovirus, influenza A & B, parainfluenza 1, 2, & 3 and respiratory syncytial virus) and *Mycoplasma pneumoniae* [13]. The viruses and *M*. *pneumoniae* were identified directly from the specimens by specific antibodies using in-house developed enzyme immunoassay. Specimens were also inoculated into 96-well microwell cell cultures for virus isolation, spun at 1000×g for 1hr at 35°C and incubated for 5–6 days at 37°C [14].

#### University Medical Center Freiburg (Freiburg, Germany)

Nasopharyngeal swab or bronchoalveolar lavage samples were collected as part of routine clinical testing. RSV was identified using the ID-Tag RVP test (Luminex, Austin, TX, USA). The ethics policy of the hospital allows for left-over specimens to be used for investigational purposes as long as they are de-identified.

#### Fondazione IRCCS Policlinico San Matteo (Pavia, Italy)

Approval for the study was obtained from the local Ethics Committee, and informed consent was obtained from patients or their parents. Nasopharyngeal aspirates were tested by cell culture, direct fluorescent antibody (DFA) staining, and quantified by real time RT-PCR [15].

#### Department of Medical Virology, University of Pretoria (Pretoria, South Africa)

Nasopharyngeal aspirates were submitted for routine diagnosis from patients with lower respiratory tract infection, hospitalized in the Kalafong and Steve Biko Academic hospitals to the Tshwane National Health Laboratory Service laboratory, Department Medical Virology University of Pretoria by direct immunofluorescent assay followed by confirmation by RT-PCR, as described previously [11]. Viruses were subsequently amplified in tissue culture for the purpose of this study. Characterization of strains by sequencing was approved and monitored by the human ethics committee, University of Pretoria (25/2006).

#### Laboratory Microbiology, Onze Lieve Vrouw Ziekenhuis (Aalst, Belgium)

Samples were collected as nasopharyngeal aspirates as part of routine clinical testing. RSV was detected using real-time RT-PCR [16]. The ethics policy of the hospital allows for left-over specimens to be used for investigational purposes as long as they are de-identified.

#### Vanderbilt Vaccine Clinic (Nashville, TN, USA)

Virus sequences were derived from specimens collected prospectively over a 20-year period from 1982–2001 in the Vanderbilt Vaccine Clinic, as previously described [17]. Nasal wash specimens were collected from children <5 years of age with acute upper or lower respiratory illness. Nasal washes were tested by cell culture, DFA staining, and quantified by real-time RT-PCR.

#### The Midwest Respiratory Virus Program, Children’s Hospital of Wisconsin, and Dynacare Laboratories (Milwaukee, WI, USA)

Nasopharyngeal swab samples were collected with informed consent under an IRB protocol or through routine clinical diagnosis and de-identified. RSV was identified by an in-house real-time RT-PCR.

Samples from the above locations where all frozen at -20°C to -85°C and held frozen until shipped to the Midwest Respiratory Virus Program for further analysis. Some of the viruses were amplified in tissue culture by inoculation in Hep-2 cells for 3–5 days prior to use in this study.

### Sanger Sequencing of RSV Genomes

Two sets of 96 pairs of degenerate RSV-specific primers tiled across the entire viral genome were designed from the consensus sequences of either three RSV-A or three RSV-B reference genomes (RSV-A accessions: RSVA/WI/629–4071/98 (JF920065), RSVA/WI/629–9/06–07 (JF920070) and RSVA/WI/629-Q0282/10 (JF920054); RSV-B accessions: RSVB/WV/14647/85 ATCC-VR1400 (unpublished), RSVB/WI/629–5B/06–07 (JN032115) and RSVB/WI/629-Q0306/10 (JN032121)) using a PCR primer design pipeline developed at the J. Craig Venter Institute (JCVI) (S1 Text and S2 Text)[18]. The primer configuration was such that produced tiled short-amplicons with an average length of 650 bp, 150 bp overlap, and with at least two-fold amplicon coverage at every base. Each primer contains a M13 sequence tag at the 5’ end used for Sanger sequencing.

Viral RNA was extracted from 100–200 μl of the samples using the ZR-96 Quick RNA(TM) kit (Zymo Research Corp., Irvine, CA, USA), following manufacturers recommendations or from 100–400 μl of the samples using the NucliSENS easyMag (bioMerieux, Inc., Durham, NC, USA) with elution in 25 μl. RNA from each sample was subjected to RT-PCR using a QIAGEN One-step kit (QIAGEN, Hilden, Germany) with four pairs of primers specific to either RSVA or B to determine the RSV type. Then, RT-PCR was performed to produce 650 bp amplicons from each of the 96 primer pairs from one of the two RSV-specific sets, A or B. Excess primers and dNTPs were removed by treatment with Exonuclease I (New England Biolabs, Ipswich, MA, USA) and shrimp alkaline phosphatase (Affymetrix, Santa Clara, CA, USA): 37°C for 60 min, followed by incubation at 72°C for 15 min, and then subjected to Sanger sequencing with M13 primers.

Sequencing reads were trimmed to eliminate amplicon primer and low-quality sequences, and assembled with Minimus, a program from the AMOS project [19]. Draft assemblies were evaluated with an in-house software, CLOE (Closure Editor, http://cloe.sourceforge.net), and targeted PCR-based sequencing reactions were conducted to close gaps and improve sequence coverage. Curated assemblies were validated and annotated with the viral annotation software VIGOR [20] and predicted genes were subjected to manual inspection and quality control before submission to GenBank.

### Sequencing of RSV Genomes by NextGen Technology

RSV isolates that could not be completely sequenced by Sanger were processed using JCVI’s Next Generation Sequencing Pipeline. Extracted genomic RNA was reverse transcribed with RSV-specific degenerate primers scattered along the RSV genome at intervals of approximately 4 kb using SuperScript III (Thermo Fisher Scientific, Waltham, MA, USA) (S1 Table). The resulting cDNA was used to generate a set of ~4 kb PCR-amplicons encompassing the entire RSV genome. PCR reactions were carried out with Accuprime (Thermo Fisher Scientific) using pairs of degenerate primers selected from the two pools of 96 primer pairs used for the Sanger sequencing pipeline. Each amplicon was gel purified and then simultaneously amplified and bar-coded using a modified sequence-independent single-primer amplification (SISPA) approach [21]. A pool of these and other viral samples were used to construct Illumina and 454 paired-end libraries and sequenced on their respective platforms. After sequencing, reads from each sample were sorted by barcode and trimmed to eliminate low quality regions as well as SISPA hexamer primer and barcode sequences. The Illumina and 454 reads were then assembled de novo using the clc_novo_assemble program (QIAGEN). The resulting contigs were then used to pick the best reference RSV genome, which was then used for a reference-based assembly using the clc_ref_assemble_long program. The assembled sequences were annotated using VIGOR as described above.

### Recombinant Analysis

All RSV genome sequences available in GenBank as of 9/24/2013 were downloaded and combined with the genomes produced in this study. Genomes from mutant RSV strains were excluded (AF013255, AF035006, U39661, U50362, U50363, and U63644). The genomes were separated by RSV subtype and aligned using MAFFT [22]. Each alignment was checked for recombination using the RDP, GENECONV, Chimaera, MaxChi, BootScan, SiScan, and 3Seq algorithms in RDP4 [23]. In order for a recombination event to be considered real it had to be detected by at least three of the algorithms. For the RSVA alignment there was a group of 14 genome sequences (GU591758—GU591771) from a single study that produced an excessive number of recombination events, as observed in a previous study [24]. These genomes were removed and the analyses were repeated. Any recombination events found were considered to most likely be sequencing errors and not true recombination. Therefore, the minor sequence in each recombinant was removed from these sequences and excluded from subsequent analyses.

### Positive Selection

All sequences available for each RSV gene were downloaded from GenBank and separated by RSV type. Sequences less than 200 base pairs or from a patent were excluded. Sequences were aligned using MAFFT and were trimmed to only the coding sequence. Coding sequences less than 50 base pairs were removed and for some genes longer sequences were removed because of non-overlapping partial sequences not aligning properly (<600bp for RSVA N gene, <500bp for RSVA F gene, and <650bp for RSVA and B G gene). Positive selection was determined for each alignment using the SLAC, FEL, and FUBAR algorithms available on the Datamonkey webserver [25–28]. Sites were only considered positive if they met the cutoff criteria for at least two of the algorithms, that is a p-value of less than 0.05 for SLAC and FEL and posterior probability of greater than 0.95 for FUBAR.

### Phylogenetic Analyses

The set of whole genome sequence alignments resulting from the recombination analysis (RSVA: 105 sequences, RSVB: 70 sequences), alignments of the full G gene CDS that have collection dates available in GenBank (RSVA: 181 sequences, RSVB: 128 sequences), and alignments of the G gene second hypervariable region for both RSVA and RSVB (RSVA: 1117 sequences, RSVB: 750 sequences) were used for the phylogenetic analyses with the Bayesian method of phylogenetic inference in BEAST v1.8.0 and the maximum likelihood method in MEGA 6.05 [29,30].

For the partial CDS alignments of the second hypervariable region in the G gene, there were many duplicate sequences. Duplicate sequences were removed with only one instance of each sequence left for each country for each year. Alignments were imported into BEAUti v1.8.0, which was used to generate xml files for use in BEAST v1.8.0. Collections dates were used to assign tip dates. A general-time reversible (GTR) model of nucleotide substitution was used with a gamma-distributed (Γ) rate variation among sites, with a proportion of invariant sites. An uncorrelated lognormal (UCLN) relaxed molecular clock was used with a flexible Bayesian skyline tree prior.

For the full CDS analysis the same model was used without a proportion of invariant sites. BEAST was used to perform a Bayesian MCMC analysis for the genome and full CDS alignments. The MCMC chain length was 250 million for the RSVA genomes, 50 million for the RSVB genomes, 150 million for the RSVA full G CDS, and three runs of 150 million for RSVB full G CDS that were combined using LogCombiner v1.8.0. The Markov chain was sampled 10,000 times for each run. BEAST results were analyzed using Tracer v1.6 and summary trees were produced using TreeAnnotator v1.8.0. Maximum likelihood trees were inferred for the genome, full CDS, and partial CDS alignments with MEGA 6.05 using the GTR model with gamma-distributed rate variant among sites. Trees were visualized and annotated in FigTree v1.4.0.

To compare the evolutionary rates between genes we extracted the complete CDS sequences for each of the 12 genes from the whole genome data sets. Alignments for each CDS were made using MAFFT. Bayesian MCMC analysis was performed with BEAST as described above for the full G CDS. A chain length of 50 million was used for each CDS sampling a total of 10,000 times. Results were analyzed using Tracer v1.6.

## Results and Discussion

Of the 100 RSV samples or isolates we attempted to sequence we were able to sequence genomes from 57, resulting in 34 RSVA and 23 RSVB genomes. Of these, 11 genomes were not completely closed and contained at least one gap. These sequences could not be closed due to insufficient sample for additional sequencing. We suspect that the samples that could not be completely sequenced had insufficient viral nucleic acids due to low viral load or possible sample degradation. It is unlikely that we were not able to sequence these viruses due to genetic variability because the number of primers used should have at least obtained partial genome sequences if there was sufficient nucleic acid. We did not sequence the 5’ and 3’ termini of the genomes, so the genomes contain incomplete 5’ and 3’ non-coding regions. Since many of these sequences (41/57) were derived directly from patient specimens they will not contain mutations that can be selected for during growth in tissue culture. For those that had been grown in tissue culture we sequenced the lowest passage available (three passages or less) for sequencing to minimize the number of culture-derived mutations. Information on the sequenced viruses can be found in Table 1 and all sequences can be retrieved from GenBank using the bioproject id PRJNA73049.

**Table 1:** Information on the RSV Strains Sequenced.

Accession Number	Strain Name	Base Pairs	Gaps (bp)
KF826827	RSVA/Homo sapiens/ARG/159/2004	15209	-
KF826828	RSVA/Homo sapiens/ARG/162/2004	15191	-
KF530260	RSVA/Homo sapiens/ARG/170/2005^a^	14980	-
KF826838	RSVA/Homo sapiens/ARG/177/2006	15189	-
KF826841	RSVA/Homo sapiens/ARG/190/2007	15194	-
KF826846	RSVA/Homo sapiens/ARG/202/2008	15142	-
KF826847	RSVA/Homo sapiens/AUS/248/2007^c^	15179	-
KF826848	RSVA/Homo sapiens/AUS/249/2007^c^	15190	-
KF530261	RSVA/Homo sapiens/DEU/106/2008^a^	15100	-
KF826830	RSVA/Homo sapiens/DEU/107/2009	15186	-
KF826831	RSVA/Homo sapiens/DEU/108/2009	15195	-
KF826854	RSVA/Homo sapiens/ITA/119/2009	15192	-
KF826832	RSVA/Homo sapiens/ITA/120/2009	15176	-
KF826833	RSVA/Homo sapiens/ITA/121/2009	15129	-
KF826855	RSVA/Homo sapiens/ITA/123/2009	15163	-
KF826856	RSVA/Homo sapiens/ITA/125/2009	15188	-
KF826826	RSVA/Homo sapiens/MEX/23/2004	15197	-
KF826816	RSVA/Homo sapiens/MEX/25/2005	14948	1215
KF826836	RSVA/Homo sapiens/MEX/26/2006	15165	-
KF826837	RSVA/Homo sapiens/MEX/27/2006	15194	-
KF826840	RSVA/Homo sapiens/MEX/29/2007	15106	-
KF826817	RSVA/Homo sapiens/MEX/43/2009	14744	117
KF530268	RSVA/Homo sapiens/MEX/59/2007^a^	15128	-
KF826852	RSVA/Homo sapiens/USA/629–1/2007^c^	15167	-
KF826850	RSVA/Homo sapiens/USA/629–11–1/2008^c^	15193	-
KF530263	RSVA/Homo sapiens/USA/629–4/2007^a^ ^,^ ^c^	15118	1279
KF826823	RSVA/Homo sapiens/USA/629–4360/1998	15197	-
KF826824	RSVA/Homo sapiens/USA/629–4392/1998	15200	-
KF826821	RSVA/Homo sapiens/USA/629–8–2/2007^c^	15177	-
KF530269	RSVA/Hep2_lab/USA/629-Q0030_RSV60/2009^a^ ^,^ ^c^	15049	-
KF826849	RSVA/Hep2_lab/USA/629-Q0115_RSV89/2010^c^	15182	-
KF530267	RSVA/Homo sapiens/ZAF/323/2007^a^ ^,^ ^b^ ^,^ ^c^	15078	1518, 530, 1797, 130, 1078
KF530258	RSVA/Homo sapiens/ZAF/324/2007^a^ ^,^ ^b^ ^,^ ^c^	15123	592
KF530264	RSVA/Homo sapiens/ZAF/332/2008^a^ ^,^ ^b^ ^,^ ^c^	15039	1002, 670, 290, 174
KF826839	RSVB/Homo sapiens/ARG/187/2006	15278	-
KF826842	RSVB/Homo sapiens/ARG/195/2007	15269	-
KF826845	RSVB/Homo sapiens/ARG/201/2008	15147	-
KF530265	RSVB/Homo sapiens/DEU/111/2009^a^	14673	258, 670
KF826853	RSVB/Homo sapiens/DEU/114/2008	15183	-
KF530266	RSVB/Homo sapiens/DEU/115/2008^a^	15064	-
KF826834	RSVB/Hep2_lab/ITA/126_RSV78/2009^c^	15218	-
KF826835	RSVB/Hep2_lab/ITA/127_RSV79/2009^c^	15238	-
KF530262	RSVB/Homo sapiens/ITA/128/2009^a^	15045	-
KF826857	RSVB/Homo sapiens/ITA/129/2009	15278	-
KF826858	RSVB/Homo sapiens/ITA/130/2009	15233	-
KF826859	RSVB/Homo sapiens/ITA/131/2009	15263	-
KF826818	RSVB/Homo sapiens/ITA/132/2009	14730	207, 1197, 1383, 1456, 687
KF826819	RSVB/Homo sapiens/ITA/133/2009	15200	926, 737, 388
KF826820	RSVB/Homo sapiens/ITA/134/2009	15181	54
KF826860	RSVB/Homo sapiens/ITA/135/2009	15249	-
KF826825	RSVB/Homo sapiens/MEX/20/2004	15259	-
KF826829	RSVB/Homo sapiens/MEX/24/2005	15215	-
KF826843	RSVB/Homo sapiens/MEX/51/2008	15280	-
KF826844	RSVB/Homo sapiens/MEX/62/2008	15218	-
KF826851	RSVB/Homo sapiens/USA/629–24/2007^c^	15279	-
KF826822	RSVB/Homo sapiens/USA/629–5/2007^c^	15269	-
KF530259	RSVB/Homo sapiens/ZAF/319/2006^a^ ^,^ ^b^ ^,^ ^c^	15157	308

^a^ These sequences were sequenced by Next-Gen sequencing.

^b^ Partial G CDS sequences have previously been published for these samples with different strain names. Accession numbers for these sequences are HQ711732, HQ711709, HQ711688, and HQ711801 [11].

^c^ These sequences were produced from viruses isolated in tissue culture.

### Recombination

We identified eight recombination events in six RSVA genomes and two events in two RSVB genomes. Five of the RSVA genomes (JF920059, JF920061, JF920064, JF920067, and JF920068) and one RSVB genome (JN032121) were from a previous study from our group [31], and were identified as potential recombinants in a previous study [24]. Upon reexamining the sequencing reads we found that the reads contributing to the minor components for these genomes appeared to be from a different RSV strain due to mismatches in overlapping reads near the predicted recombination breakpoints. In one genome (JF920068) we found additional reads in locations not detected by the recombination analysis that appeared to be from a different strain. These reads were removed and the genomes were reassembled. Since we suspected that all predicted recombination events were sequencing errors the predicted recombinant regions from the remaining two genomes (JX015495 and JX576753) were also removed. These results highlight the importance of running a recombination analysis when performing genome sequencing even when one does not expect true recombination events.

### Entropy Plots of the RSVA and B Protein Sequences

**Fig 1:**
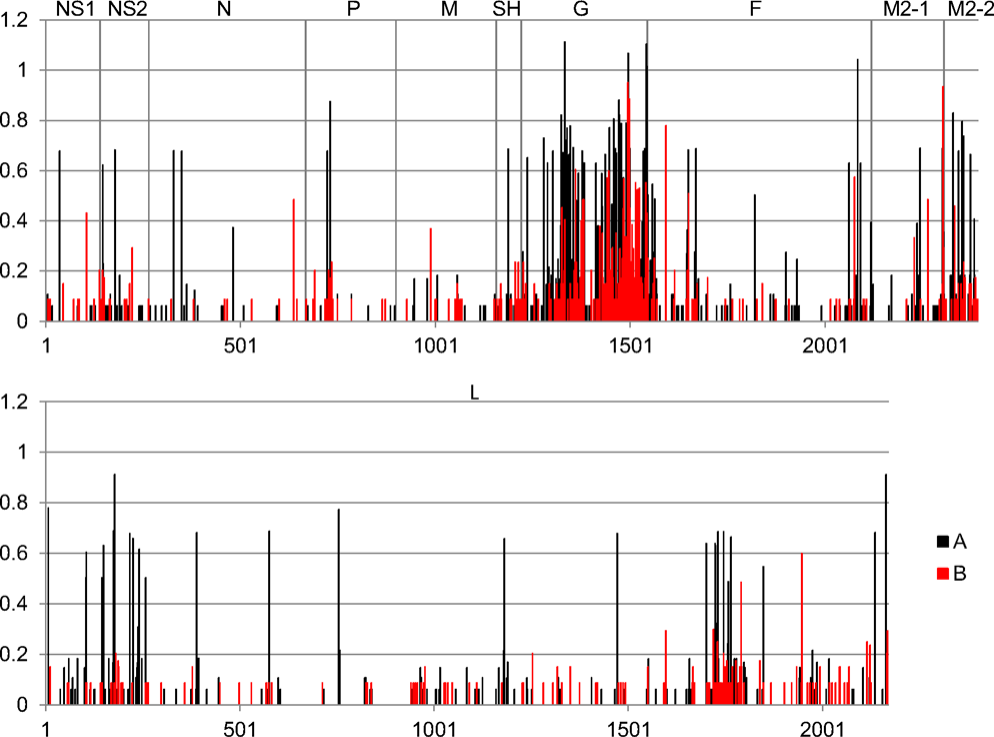
Protein Entropy Plots. This is an entropy plot of the concatenated predicted protein sequences of all of the viruses sequenced in this study. Entropy values were calculated using BioEdit 7.0 and the plot was generated using Microsoft Excel. Black bars are for RSVA sequences and red bars are for RSVB sequences. The higher the bar is the greater the variation at that position in the protein sequence. Across the top of the plot are listed the abbreviated protein sequence names in the order in which the CDS sequences for the proteins appear in the genome.

From the whole genome alignments the CDS sequences for each RSV gene were concatenated into a single sequence and then translated into predicted protein sequences. The entropy values for each amino acid position were calculated using the Entropy (H(x)) plot function in BioEdit 7.0 (Fig. 1). From these plots it is clear that the G protein is the most variable in both RSVA and B. The high variability of the G gene/protein makes it a good target for evolutionary analyses, which has been the primary goal of most RSV sequencing studies. Therefore, there is a large amount of sequence data for this gene. For the remaining genes/proteins there is significantly less sequence data available. These more conserved regions of the genome are critical for the development of robust diagnostics that continue to detect currently circulating strains.

### Positively Selected Sites

We used the SLAC, FEL, and FUBAR algorithms on the Datamonkey webserver to identify potential positively selected sites for each of the coding regions in RSV [27,28]. These programs take alignments of coding sequences and use algorithms to predict if codon sites are under different types of selection. In general, positions under positive selection show a shift over time from one amino acid to another presumably due to a fitness benefit. Positions under negative selection tend to rapidly remove amino acid variants because they may be deleterious. There were multiple positively selected sites predicted in the G and F coding regions (Table 2). These results are not surprising considering both of these genes produce surface glycoproteins that likely face selective pressure from the host immune system. The predicted positively selected sites in the fusion protein were located in the N-terminal signal peptide and the C-terminal heptad repeat. Two additional sites (553 and 573) had initially been predicted in the RSVB F gene cytoplasmic tail region, but upon close inspection they were found to be artifacts from primer sequences used in previous studies [32,33]. In the attachment glycoprotein, predicted positively selected sites were located in the N-terminal cytoplasmic domain and the mucin-like regions. It is unclear what would be driving positive selection in the few sites that are located on the cytoplasmic regions of these proteins. None of the other RSV coding regions had sites that met the criteria of having two of the three algorithms predicting positive selection with the cutoffs used in this study. Since the criteria we used were fairly conservative our results do not eliminate the possibility that there are sites under positive selection in these coding regions.

**Table 2:** Sites under positive selection in RSVA and RSVB.

Type	Gene	Site	SLAC		FEL		FUBAR		Reference Position^a^
			dN-dS	p-value	dN-dS	p-value	dN-dS	Post. Pr.	
RSVA	G	4	3.237	0.042	1.091	0.002	0.779	0.994	4
		124	0.752	0.019	0.433	0.043	0.176	0.877	**124**
		161	0.338	0.233	0.346	0.009	0.202	0.957	**161**
		162	0.474	0.129	0.35	0.006	0.212	0.969	162
		247	1.333	0.002	1.162	0.005	0.847	0.998	**244**
		258	0.405	0.175	0.453	0.003	0.239	0.977	**255**
		301	1.686	0.001	0.807	0.062	0.697	0.98	**274**
		313	1.879	<0.001	0.776	0.002	0.637	0.994	**286**
		317	2.885	0.003	0.834	0.017	0.716	0.989	**290**
		324	2.009	0.15	1.751	0.004	2.034	0.998	**297**
	F	19	5.37	0.083	1.931	0.029	1.025	0.964	19
		518	6.54	0.026	0.787	0.008	0.324	0.956	518
RSVB	G	219	4.19	<0.001	1.318	0.001	0.81	1	**219**
		286	1.288	0.005	0.498	0.005	0.248	0.961	261
		292	1.571	0.011	1.043	0.003	0.806	0.996	**267**
		305	0.894	0.027	0.617	0.035	0.207	0.874	280

^a^ Position in reference sequence. Accession M74568 is the reference for RSVA and AF013254 is the reference for RSVB. Sites in bold were also predicted to be under positive selection in previous publications.

Since these algorithms were designed for sequences from divergent populations [34], Kryazhimskiy and Plotkin 2008 recommend that caution be taken when interpreting results from using these algorithms with closely related sequences belonging to the same species. Generally these algorithms use different methods to estimate the ratio of nonsynonymous changes per nonsynonymous site (dN) to synonymous changes per synonymous site (dS). Sites with a dN/dS ratio > 1 are considered to be under positive selection and those < 1 under negative selection. They showed that these assumptions may not always hold true with sequences from the same population/species and sites under positive selection may be underestimated. Therefore, it is likely that there are additional sites under positive selection that were not identified in this study and other similar studies. Despite these limitations, other studies have also tried to identify positively selected sites in RSV.

**Fig 2:**
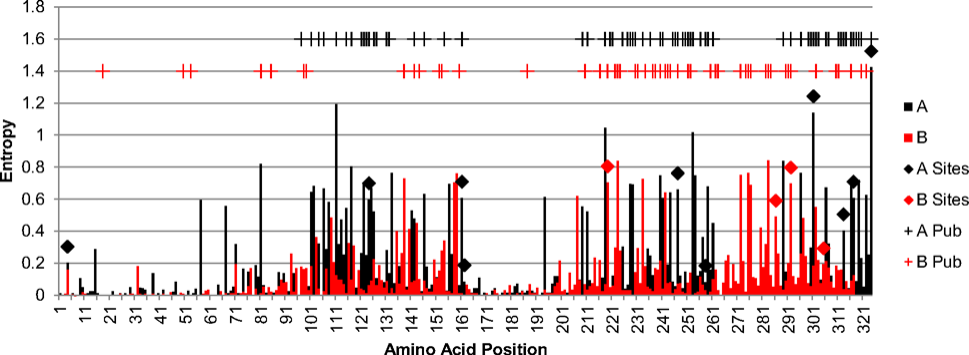
G Protein Entropy Plot and Positive Selected Sites. This is an entropy plot of the G protein sequences with positively selected sites also shown. Entropy values were calculated using BioEdit 7.0 from the alignments used for the positive selection analysis and the plot was generated using Microsoft Excel. Black bars are for RSVA sequences and red bars are for RSVB sequences. Sites predicted to be under positive selection in this study are shown with black diamonds for RSVA and red diamonds for RSVB. Near the top are shown sites predicted to be under positive selection or diversifying selection from previously published studies with black pluses for RSVA and red pluses for RSVB.

We reviewed seven previously published studies to identify which sites have previously been predicted to be under positive selection [24,33,35–39]. Most of these studies focused on the G CDS, and the majority of the positively selected sites identified in this study were also identified in one or more of the previous studies (8/10 for RSVA and 2/4 for RSVB). Across all studies most of the sites predicted to be under positive selection were identified in only one study (60% for RSVA and 74% for RSVB). Differences between studies can be attributed to differences in data sets, prediction algorithms, and cutoff criteria used. However, like the previous studies most of the positively selected sites were identified in the highly variable mucin-like regions of the G gene (Fig. 2). It has previously been demonstrated that both humans and rabbits produce antibodies to these highly variable regions [40,41]. One of these studies used linear peptides representing natural amino acid variations in the carboxy-terminal mucin-like region of the G protein to demonstrate that human serum can contain antibodies to these regions of the protein and that even a single mutation can eliminate reactivity [41]. Additionally, reactivity of the serum with the peptides was dependent on the genotype of the RSV virus that infected the individual, and a mutation in one of the predicted positively selected sites (position 244 in M74568) was found to produce an antibody escape mutant [41]. Taken together these results support the concept that immunologic pressure is driving selection of mutations in these regions and that mutations in these regions play a role in the ability of RSV to re-infect individuals throughout their lives.

### Gene Start (GS), Gene End (GE), and Stop Codon Sequences

Each gene in RSV has a specific nine nucleotide “gene start” sequence (3’-CCCCGUUUA-5’) that directs transcription initiation that is almost completely conserved in all genes in both RSVA and RSVB, with only the polymerase gene (3’-CCCUGUUUU-5’) in both RSVA and RSVB and the small hydrophobic gene (3’-CCCCAUUUA-5’) in RSVB typically having a different sequence. In one of our RSVB genome sequences (KF826829) we found a mutation in the GS sequence of the SH gene resulting in 3’-CCCUAUUUA-5’ as the GS. It is unknown how this mutation affects the viability of the virus. We found two other published RSVB sequences (JX576736, JX576757) that had a C->U mutation in the same position of the GS sequences in the M and F genes. Interestingly, this mutation actually matches the base at this position of the L gene GS. This suggests that this specific mutation may be tolerated better by the transcriptional machinery than other mutations in the GS sequences. To support this suggestion, a mutagenesis study of the GS showed that this mutation had no negative impact on expression levels [42]. The only other published sequences with mutations in a GS were a temperature sensitive mutant of the RSV A2 strain (U63644) with an A->G mutation at the last position of the M2 gene GS, which was shown to be the cause of temperature sensitivity [43], and a sequence from China with two mutations in the GS of the F gene (AY198177), which appears to have been introduced by the amplification primers [44].

Each gene in RSV also ends with a less conserved sequence that directs transcription termination and polyadenylation that follows the motif (3’-UCAAUN_1–4_U_4–8_–5’) (Table 3). Unlike the GS, these GE sequences are usually different for each gene, different between RSVA and RSVB, and exhibit more strain-to-strain variation. The first five bases are conserved in most of the GE sequences, however, some variation is observed in positions three and four. In our previous genome sequencing study we identified previously unseen variations of the GE sequences [31]. Again in this study we found additional variant GE sequences highlighting the relative flexibility of these sequences, which have been shown to affect the efficiency of transcription termination which in turn affects the expression levels of downstream gene sequences [45]. Most of the variations are the result of differences in the length of the poly U tract.

**Table 3:** Gene End Sequences of RSVA and RSVB.

Gene	RSVA		RSVB	
	Sequence 3’→5’^a^	Occurrences^b^	Sequence 3’→5’	Occurrences
**NS1**	UCAAUUAUAUUUU	34	UCAAUUAUAUUUU	23
**NS2**	UCAUU-AAAUUUU	17	UCAUU-A-AUUUUU	23
	UCAAU-AAAUUUU	16		
**N**	UCAAUU—AUUUUUU	27	UCAAUU—GUUUUUU	22
	UCAAUU—AUUUUUUU	1	UCAAUU—GUUUUU	1
	UCAAUU—AUUUUU	3		
	UCAAUU—GUUUUU	2		
**P**	UCAAU—-GUUUUUUU	27	UCAUU—-GUUUUUUU	22
	UCAAU—-GUUUUUUUU	2	UCAUU—-GUUUUUUUU	1
	UCAAU—-GUUUUUU	2		
	UCAAU—-GUUUUU	1		
	UCAAU—GCUUUUUU	2		
**M**	UCAAUU—AUUUUUU	12	UCCAUUU-AUUUU	22
	UCAAUU—AUUUUU	19	UCUAUUU-AUUUU	1
	UCAAUU—AUUUUUUU	1		
**SH**	UCAAUU-AAUUUUU	26	UCAAU-AAAUUUUU	21
	UCAAUU-AAUUUUUU	6	UCAAU-AAAUUUU	2
**G**	UCAGU—AAUUUUU	29	UCAAU-AAAUUUUU	20
	UCAGU—AAUUUUUU	2	UCAAU-AAGUUUUU	3
	UCAAU—AAUUUUU	2		
**F**	UCAAU-AUAUUUU	29	UCAAU-GUAUUUUU	21
	UCAAU-AUAUUUUU	1	UCAAU-AUAUUUUU	2
	UCAAU-AUAUUUUUU	2		
	UCAAU-AUAUUUUUUU	1		
	UCAGU-AUAUUUU	1		
**M2**	UCAAU-AAAUUUU	30	UCAAU-AGAUUUU	20
**L**	UCAAU-AAAUUUU	31	UCAAU—AAUUUUUU	21
			UCAAU—AAUUUUU	1
			UCAGU—AAUUUUUU	1

^a^ Underlined sequences were identified previously. Dashes were added so the U-tracts line up.

^b^ These numbers only include sequences from this study.

Stop codon locations are generally well conserved in both location and sequence for most of the genes in RSV. The gene with the most variable stop codons is the G gene for which variation in stop codons is common resulting in proteins of varying lengths [46]. The G gene stop codon positions of sequences produced in this study matched the positions of those in previously published sequences. These positions were codons 298 and 299 in RSVA and 289, 292, and 296 in RSVB using accessions M74568 and AF013254 as reference sequences. In RSVA both stop codons are utilized with about equal frequency, while in RSVB the stop codon at position 289 is utilized most frequently in the BA clade which represents the majority of recent isolates. In one of our RSVB sequences (KF826839) we identified a premature stop codon in the SH gene resulting in a reduction in the expected protein size from 65 aa to 56 aa. We were able to grow this virus in culture and we confirmed by sequencing the SH gene that the isolate also contained the mutation. The SH protein is a viroporin that has been shown to form a pentameric ring in the cell membrane that functions as a cation channel [47]. The missing 9 aa would be part of an extended loop structure of the extracellular C-terminal domain. It is not known if these amino acids are important for protein function.

### Evolutionary Rates

**Fig 3:**
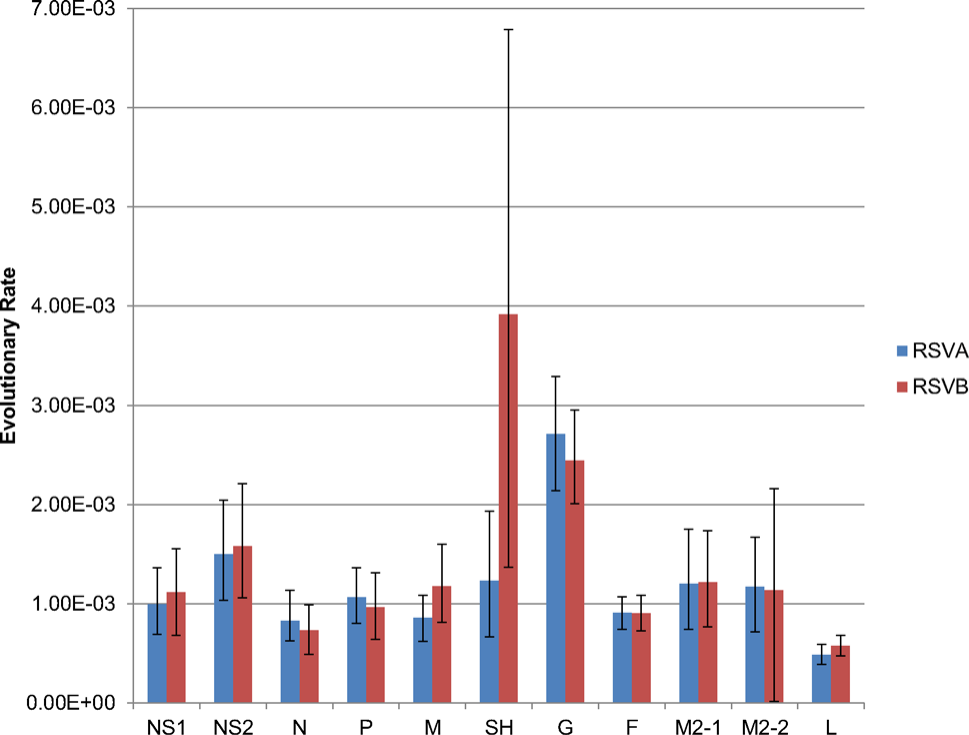
CDS Evolutionary Rates. This plot shows the estimated evolutionary rates for each CDS in RSVA and RSVB. Error bars represent the 95% HPD values. Rates were determined using BEAST v1.8.0 with the GTR model of nucleotide substitution, a gamma-distributed rate variation among sites, an uncorrelated lognormal relaxed molecular clock, and a flexible Bayesian skyline tree prior. Each CDS was ran with a chain length of 50 million and sampled 10,000 times.

The evolutionary rates of the whole genome sequences were 6.72 × 10^-4^ substitutions/site/year [95% HPD 5.61 × 10^-4^ to 7.91 × 10^-4^] for RSVA and 7.69 × 10^-4^ substitutions/site/year [95% HPD 6.81 × 10^-4^ to 8.62 × 10^-4^] for RSVB. The evolutionary rates based on the G CDS were 1.86 × 10^-3^ substitutions/site/year [95% HPD 1.64 × 10^-3^ to 2.08 × 10^-3^] for RSVA and 2.43 × 10^-3^ substitutions/site/year [95% HPD 2.05 × 10^-3^ to 2.84 × 10^-3^] for RSVB. Evolutionary rates varied among individual genes, but all remained within the range of 4.22 × 10^-4^–3.73 × 10^-3^ substitutions/site/year (Fig. 3), similar to what has been observed previously [38].

### Phylogenetic Trees

We inferred maximum likelihood (ML) and maximum clade credibility (MCC) trees for the whole genome and full G gene CDSs and only ML trees for the partial G gene CDSs. For RSVA, the partial G gene CDS covered nucleotides 677–891 (216 nucleotides) for sequence JN257702 and for RSVB, it covered nucleotides 640–853 (214 nucleotides) for sequence AY353550. The size of the CDS fragment varied due to various insertions. This region was reported most frequently in previous RSV phylogenetic studies and includes the 72 nucleotide insertion found in the recent RSVA ON1 genotype and 60 nucleotide insertion found in the RSVB BA genotype. We found that the trees produced by both methods for the whole genome and full G gene CDS sequences were similar, and that there was good agreement between the whole genome and full G gene CDS trees. However, there were noticeable topological differences in the partial G gene CDS trees relative to the whole genome and full G gene CDS trees. These rearrangements were most evident in RSVB, for which sequences belonging to SAB3 genotype that lack the 60 nucleotide insertion were found to cluster within the BA genotype only in the partial CDS tree. These results suggest that both the whole genome and full G gene CDS are suitable for evolutionary analyses, but the partial G CDS alone may produce misleading results.

### RSVA Phylogenetic Analysis

**Fig 4:**
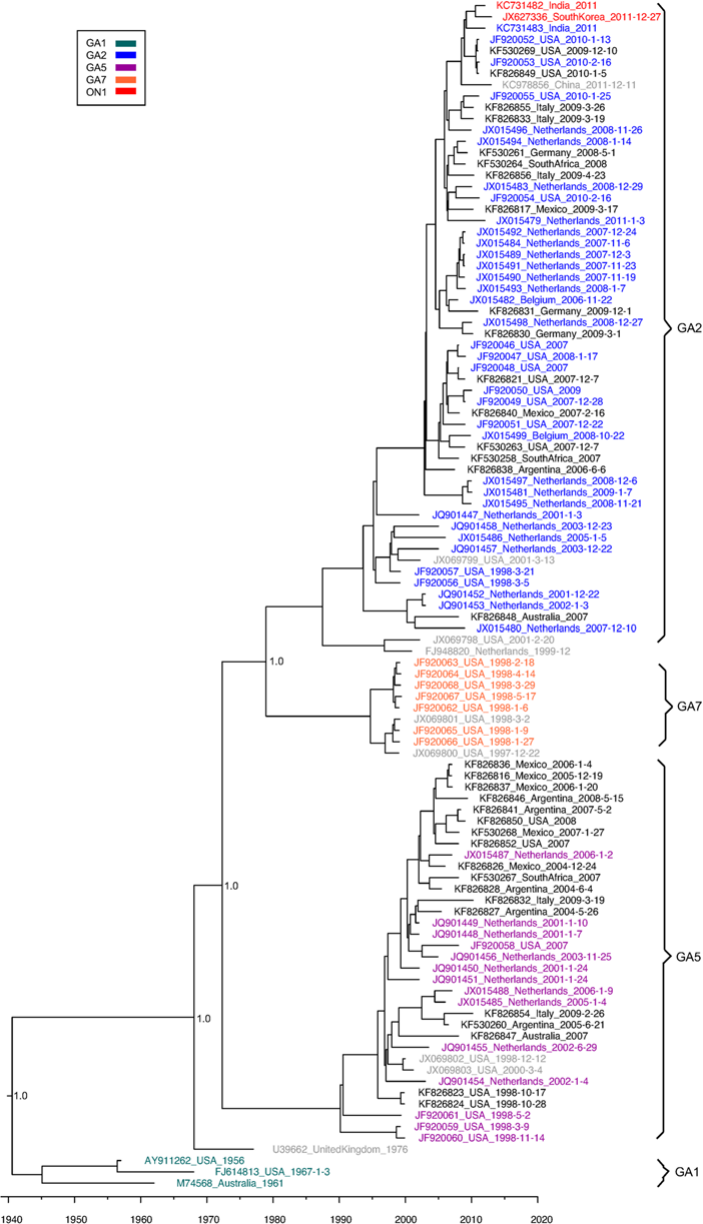
Tree of RSVA Genome Sequences. This is a maximum clade credibility tree of RSVA genome sequences generated in this study and retrieved from GenBank. Tip times correspond to date of collection with the scale axis across the bottom showing the years. Tip labels show the accession number, country of isolation, and collection date. The labels are color coded with black for sequences from this study (FTS), grey for sequences with an undetermined genotype (UND), and the remaining colors corresponding to previous published genotypes as show in the key in the upper left corner. Brackets highlight the major clades. Bayesian posterior probabilities are shown for key nodes.

The MCC tree inferred for the RSVA genome (Fig. 4) includes viruses that have previously been characterized as belonging to one of the defined genotypes: GA1, GA2, GA5, GA7, and ON1. The full CDS tree (S1 Fig.) also included viruses from genotypes NA1 and CB-A, while the partial CDS tree (S2 Fig.) includes viruses from NA2 and a single virus from GA3 and SAA1. We found that the majority of sequences in our data set that were collected globally over the past 10 years belong to one of two clades of RSVA. The larger of these clades encompasses the GA2, NA1, NA2, CB-A, and ON1 genotypes, with the oldest virus dating back to 1994. Different studies have used different reference sequences for defining genotypes, making it difficult at times to distinguish between the genotypes. Therefore, we found genotypes not to be particularly useful to characterize viral diversity in our study, and instead will refer to this large clade simply as the GA2 clade based on the oldest genotype found within it. The ON1 genotype contains a 72 nucleotide insertion in the second variable region of the G gene and includes sequences from 2012–2013 from Canada, Italy, Germany, China, Japan, South Korea, South Africa, Croatia and India [6,9]. The RSVB BA genotype also has a large insertion in the same region (60 nt) that emerged in the late 1990s and then rapidly spread globally. It will be interesting to see if the ON1 genotype becomes the predominant RSVA genotype as the BA genotype did for RSVB.

The second predominant clade contains sequences from the GA5 genotype, with the oldest one from 1993. Of the 34 RSVA genomes sequenced in this study, 16 belonged to the GA2 clade and 18 belonged to the GA5 clade. The other two clades represent the GA1 and GA7 genotypes. The GA1 clade contains the oldest identified RSVA viruses (1956–1967), two viruses from our previous study (JF920069 and JF920070, not shown due to potential contamination), and a group of viruses from Iran from 2008–2009 (only in the partial CDS tree). One of the Iran viruses (GU339399) is a 100% match to the A2 strain from 1961 (M74568) and the remaining strains are much less divergent than one would expect given the time frame [48,49]. Therefore, we suspect that this could be a re-introduction of a laboratory strain into the population and is now circulating in Iran. Since we retrieved the sequences used in this study, another study has been published showing additional viruses from this clade isolated in 2012 and 2013 in Iran, providing further evidence that the this virus is indeed currently circulating and not simply a lab/sequencing error [50]. For the GA7 clade only one virus is from after 2002 (JX256946) and is a 100% match to a 1994 virus (JX256947) from the same study suggesting possible contamination [51]. The relatively low number of GA7 sequences suggests that this clade may no longer be circulating or present infrequently enough that it is rarely detected.

### RSVB Phylogenetic Analysis

**Fig 5:**
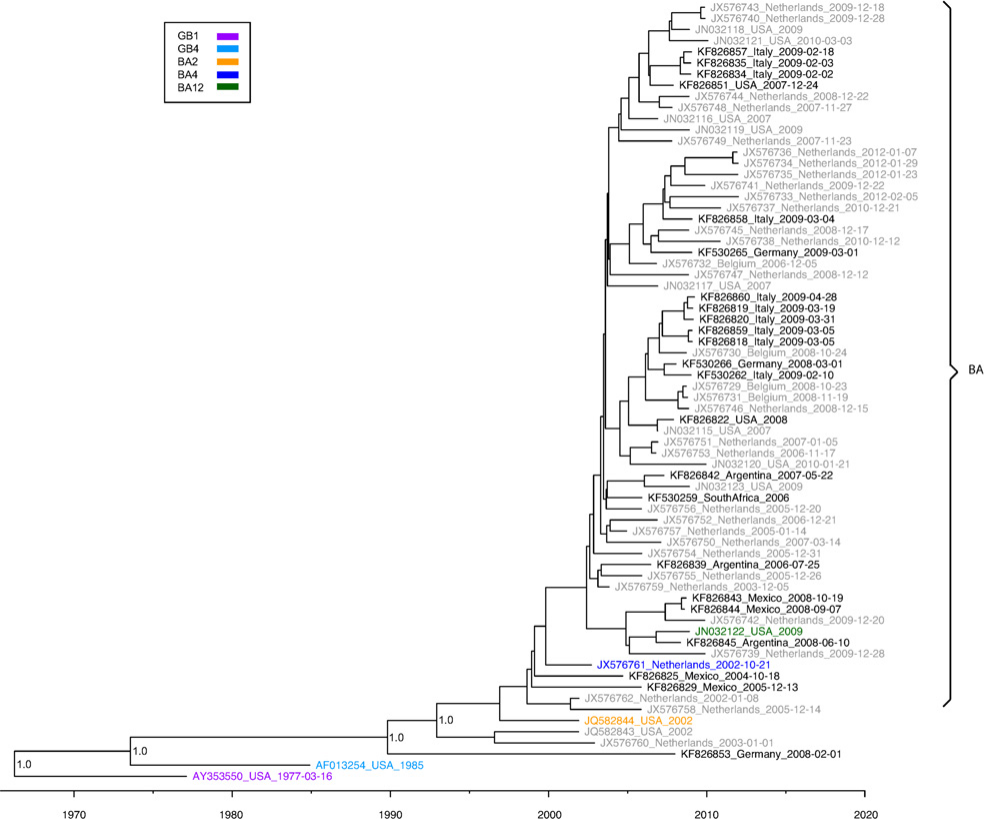
Tree of RSVB Genome Sequences. This is a maximum clade credibility tree of RSVB genome sequences generated in this study and retrieved from GenBank. Tip times correspond to date of collection with the scale axis across the bottom showing the years. Tip labels show the accession number, country of isolation, and collection date. The labels are color coded with black for sequences from this study (FTS), grey for sequences with an undetermined genotype (UND), and the remaining colors corresponding to previous published genotypes as show in the key in the upper left corner. Brackets highlight the major clades. Bayesian posterior probabilities are shown for key nodes.

The RSVB genome tree (Fig. 5) includes viruses that had previously been described as belonging to genotypes GB1, GB3, GB4, SAB3, and BA. The full CDS tree (S3 Fig.) also includes viruses from genotypes SAB1 and SAB2 while the partial CDS tree (S4 Fig.) includes viruses from SAB4, GB2 and GB12. Since the RSVB BA genotype emerged in the late 1990s it spread globally and became the predominant genotype [52]. Matching this data, we found the BA clade to represent the largest proportion of sequences in this study, with 22 of the 23 viruses we sequenced belonging to this clade. Many studies have divided members in the BA clade into as many as 13 different genotypes. However, since different studies use different reference sequences for the genotypes and disagree on the total number of genotypes there is much overlap between genotypes, as evidenced by the partial CDS tree. For example, sequences classified as belonging to the BA4 genotype are intermixed with those reported as belonging to the BA7, BA8, BA9, and BA10 genotypes. Despite some fairly distinct clusters of sequences most of the BA viruses could not readily be divided into separate clades. Only one of the viruses (KF826853) sequenced in this study belonged to a genotype other than BA (GB3). Viruses in the GB3/SAB4 clade were identified from 2000 to 2012 in Africa, Asia, Europe, North America, and South America showing that this clade has continued to circulate globally at low levels [53]. Viruses have also been identified as recently as 2011 for the GB2 clade [54]. This shows that even though the viruses that belong to the BA clade have almost completely replaced the older clades some of the non-BA clades have continued to circulate either in distinct regions or globally at low levels.

## Conclusions

Through this work we have contributed an additional 57 RSVA and RSVB whole virus genomes to GenBank, significantly increasing the total number of available genomes for RSV. In addition, these genomes have considerable geographic diversity. By incorporating additional publically available sequences in our analyses we were able to develop a better understanding of the evolution and global circulation of RSV viruses. We found that the GA2 and GA5 clades for RSVA and the BA clade for RSVB have been the predominant RSV clades circulating globally for at least the past 10 years. Importantly, we did not observe a strong spatial structure in the tree, with evidence that both main RSVA clades co-circulate globally. These findings suggest that the evolution of RSVA and RSVB is not strongly regionalized, although some small clades were only identified in one country (e.g., GA7 in the United States). Further surveillance and sequencing of RSV is required to determine if these minor clades are still circulating in under sampled regions, as well as to know whether certain RSV lineages dominate over sustained time periods within a defined region, or fluctuate year-to-year. Our phylogenies also indicate that previously used genotype classifications may not be monophyletic, and therefore highlight the importance of developing improved nomenclature for RSV.

The majority of RSV sequencing has been focused on RSV evolution and therefore has primarily targeted only a partial region of a single RSV gene. Until recently, this meant that there were only very few sequences for the conserved regions of RSV, which are critical for the development of robust diagnostics. Since viruses continuously evolve, the availability of more whole virus genome sequences from recent isolates representing the diversity of the circulating RSV population will aid in the development of reliable diagnostics and monitoring for changes in areas targeted by current and future RSV therapeutics.

## Supporting Information

S1 Fig Tree of RSVA Attachment Glycoprotein CDS Sequences. This is a maximum clade credibility tree of RSVA attachment glycoprotein CDS sequences generated in this study and retrieved from GenBank. Tip times correspond to date of collection with the scale axis across the bottom showing the years. Tip labels show the accession number, country of isolation, and collection date. The labels are color coded with black for sequences from this study (FTS), grey for sequences with an undetermined genotype (UND), and the remaining colors corresponding to previous published genotypes as show in the key in the upper left corner. Brackets highlight the major clades. Bayesian posterior probabilities are shown for key nodes.
(PDF)

S2 Fig Tree of RSVA Attachment Glycoprotein Partial CDS Sequences. This is a maximum likelihood tree of RSVA attachment glycoprotein partial CDS sequences corresponding to the second hyper variable region generated in this study and retrieved from GenBank. Tip labels show the accession number, country of isolation, and collection date. The labels are color coded with black for sequences from this study (FTS), grey for sequences with an undetermined genotype (UND), and the remaining colors corresponding to previous published genotypes as show in the key in the upper left corner. Brackets highlight the major clades.
(PDF)

S3 Fig Tree of RSVB Attachment Glycoprotein CDS Sequences. This is a maximum clade credibility tree of RSVB attachment glycoprotein CDS sequences generated in this study and retrieved from GenBank. Tip times correspond to date of collection with the scale axis across the bottom showing the years. Tip labels show the accession number, country of isolation, and collection date. The labels are color coded with black for sequences from this study (FTS), grey for sequences with an undetermined genotype (UND), and the remaining colors corresponding to previous published genotypes as show in the key in the upper left corner. Brackets highlight the major clades. Bayesian posterior probabilities are shown for key nodes.
(PDF)

S4 Fig Tree of RSVB Attachment Glycoprotein Partial CDS Sequences. This is a maximum likelihood tree of RSVB attachment glycoprotein partial CDS sequences corresponding to the second hyper variable region generated in this study and retrieved from GenBank. Tip labels show the accession number, country of isolation, and collection date. The labels are color coded with black for sequences from this study (FTS), grey for sequences with an undetermined genotype (UND), and the remaining colors corresponding to previous published genotypes as show in the key in the upper left corner. Brackets highlight the major clades.
(PDF)

S1 Table Primer Pairs Used for Next Gen Sequencing. (XLS)

S1 Text Primer Pairs Used for Sanger Sequencing of RSVA. (TXT)

S2 Text Primer Pairs Used for Sanger Sequencing of RSVB. (TXT)
