# Supplementary material for: Sequencing and Analysis of Globally Obtained Human Respiratory Syncytial Virus A and B Genomes
Source: PLoS One. 2015 Mar 20;10(3):e0120098. doi: 10.1371/journal.pone.0120098 (PMC4368745; doi:10.1371/journal.pone.0120098)

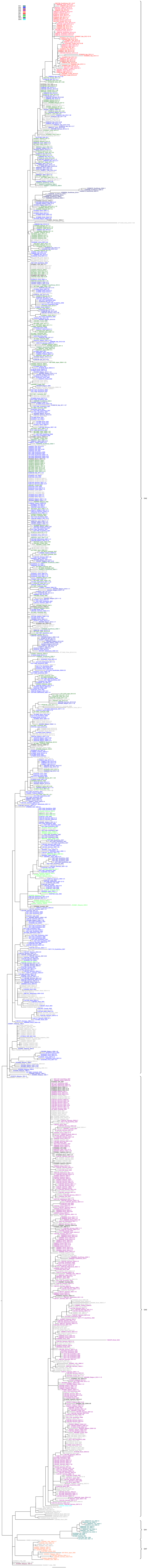

Supplement: S2 Fig — This is a maximum likelihood tree of RSVA attachment glycoprotein partial CDS sequences corresponding to the second hyper variable region generated in this study and retrieved from GenBank. Tip labels show the accession number, country of isolation, and collection date. The labels are color coded with black for sequences from this study (FTS), grey for sequences with an undetermined genotype (UND), and the remaining colors corresponding to previous published genotypes as show in the key in the upper left corner. Brackets highlight the major clades. (PDF) [file pone.0120098.s002.pdf]
